# Paediatric trauma resuscitation: an update

**DOI:** 10.1007/s00068-015-0614-9

**Published:** 2015-12-22

**Authors:** T. H. Tosounidis, P. V. Giannoudis

**Affiliations:** Academic Department of Trauma and Orthopaedic Surgery, University of Leeds, Clarendon Wing, Floor A, Great George Street, Leeds General Infirmary, Leeds, LS1 3EX UK; NIHR Leeds Biomedical Research Unit, Chapel Allerton Hospital, West Yorkshire, Leeds, LS7 4SA UK

**Keywords:** Paediatric trauma, Paediatric injury, Paediatric resuscitation, Paediatric mortality

## Abstract

**Purpose:**

Paediatric trauma is the leading cause of mortality in children. Paediatric trauma resuscitation is the first and foremost step towards a successful treatment and subsequent recovery. Significant advances have taken place in the last years in relation to this field of trauma care.

**Methods:**

In this narrative review, we attempt to summarise the recent development in the concepts of fluid resuscitation, massive transfusion, permissive resuscitation, management of coagulopathy and use of tranexamic acid, literature pertaining to implementation of transfusion protocols in the paediatric population and education related to the paediatric trauma resuscitation.

**Results/Conclusions:**

The current evidence although emerging is still sparse and high-quality studies are needed to shed more light on most of the above domains of resuscitation.

## Introduction

Trauma continues to be the leading cause of mortality in children and contemporary evidence suggests that in developed countries trauma is also the major cause of disability in the paediatric population [1, 2]. Paediatric trauma resuscitation constitutes the first of  the multiple steps that are needed to achieve full recovery of the traumatised child; a feasible goal that should always be taken into consideration given the remarkable degree of recovery observed in polytrauma children. The vast majority of literature pertaining to the acute trauma care is derived from studies in adults. Nevertheless, the paediatric trauma population represents a unique challenge due to differences in anatomy, physiology and age-specific considerations. In this narrative review of the literature, we attempt to summarise the advances that have taken place over the last 5 years in paediatric trauma resuscitation with emphasis on fluid resuscitation, massive transfusion, permissive resuscitation, management of coagulopathy and use of tranexamic acid and evidence pertaining to implementation of transfusion protocols in the paediatric population. Considering the importance and inherent difficulties in education related to paediatric resuscitation, we also present some of new evidences regarding the use of simulation in paediatric resuscitation.

### Fluid management

Fluid resuscitation necessitates a potent vascular access. It has recently been highlighted that vascular access for adequate fluid resuscitation can be a challenge in the pre-hospital setting [3, 4]. A retrospective review [5] evaluating the emergency medical service interventions including endotracheal intubation, intravenous access and fluid resuscitation demonstrated that the pre-hospital care of children was substantially deficient in comparison to the one provided to adults. In particular, compared to adults more children required venous access upon their arrival to the hospital. The authors attributed the above finding to hypovolemia at presentation and observed increased number of adverse events related to unsuccessful attempts such as bruises and hematomas.

Fluid resuscitation in the paediatric population should be physiology-driven and at the same time, respect the different aspects of age-related differences in this population. Excessive fluid resuscitation might be harmful and up-to-date there are no firm guidelines regarding the optimal resuscitation fluid volume in paediatric trauma. In a retrospective review of the practice of a designated paediatric trauma centre in Canada, Al-Sarif et al. studied the practice of fluid resuscitation and the associated complications in non-hemorrhagic blunt trauma paediatric patients. The authors concluded that the administered fluid volume was excessive and that 12 % of the 139 patients that were included in the study developed “fluid resuscitation attributable complications” such as ascites and/or pleural effusions. The authors concluded that over-resuscitation is a common and potentially harmful phenomenon in non-hemorrhagic paediatric trauma. In the same line, Edwards et al. in a recent retrospective review of Department of Defense Trauma Registry (US military) of 907 children 14 years old or younger, concluded that crystalloid-predominant resuscitation had a negative effect on mortality, hospital and intensive care length of stay and increased ventilator days. The above effect was also evident even when adjusted for age and Injury Severity Score. Interestingly, balanced component resuscitation with FFP and RPBCs or whole blood did not yield better results as well. The authors point out that further studies are needed to determine the effects of balanced resuscitation in the bleeding paediatric patients and suggest that future research should focus on delineation of appropriate physiologic triggers of resuscitation strategies based on the age of the patient.

### Massive transfusion

Massive transfusion is a strategy to deal with the bleeding critically ill trauma patient by administering large volume of blood products in a short period of time. It is a well-established practice in the adult population and over the last years it has been clearly proven beneficial for the adult trauma patient [6, 7]. Massive transfusion protocols for children are not yet fully developed [8] but this field has recently gained attention and massive transfusion protocols for the paediatric population have started emerging. Nevertheless, the vast majority of the existing studies are retrospective in nature and the level of evidence is low.

Defining massive transfusion is of paramount importance. In the adult population various definitions exist [6]. Similarly several different definitions have been created for the paediatric trauma population with none being universally accepted [9, 10]. Recently, Diab et al. [8] suggested the following dynamic definition of massive transfusion in children and neonates: “transfusion of >50 % TBV in 3 h, transfusion >100 % TBV in 24 h or transfusion support to replace ongoing blood loss of >10 % TBV per min”. Moreover, Neff et al. [11] utilised data from the Department of Defense Trauma Registry (US Military) and retrospectively reviewed 1113 paediatric (<18 years of age) trauma (combat-injured) patients that were transfused during the resuscitation process. The authors concluded that 40 ml/kg of all blood products administered at any time within the first day could identify the critically traumatised patients who are at increased risk for early and in-hospital death. Consequently, the authors considered the above cut off point as critical in defining massive transfusion in paediatric population and suggested that since this definition is irrelevant to the injury mechanism and also takes into account contemporary transfusion practices, it could be reliably used in future clinical research. Livinston et al. [12] reviewed the incidence, patients’ characteristic and outcomes of massive transfusion in a paediatric trauma cohort of 435 patients. The authors aimed to evaluate the outcomes of massive transfusion when it was used prior to the implementation of a specific protocol. Massive transfusion took place in 3 % of the patients and was correlated to poor outcome, severe injuries, higher incidence of head trauma and longer duration of hospital stay. Coagulopathy occurred more frequently when massive transfusion was implemented. The authors concluded that better coordination and attention to the correct amounts of frozen plasma, cryoprecipitate and platelets is needed when this tactic is used.

### Transfusion protocols

The evidence in relation to the implementation of massive transfusion protocols in paediatric trauma is sparse [13]. Hendrickson et al. [14] described the efficiency of the implementation of the transfusion protocol (fixed ratio of red blood cells: fresh frozen plasma:platelets). The authors compared demographics, resuscitation volumes and outcomes of patients managed with the transfusion protocol in historical control of patients that were managed before the implementation of this protocol. The authors came to the conclusion that mortality rates were not significantly altered after the implementation of the transfusion protocol. Nevertheless, they acknowledge the fact that the cross-sectional character of the study and the small number of patients enrolled constitute inherent weaknesses of their study and that better designed studies are needed to reach safe conclusions. In another recent retrospective review, Nosanov et al. [15] reported on the impact of plasma and platelet ratios on mortality of 105 paediatric and adolescent patients (<18 years) who received massive transfusion. Interestingly, no decrease in mortality was observed in patients who were transfused with higher plasma/RBC and plasma/platelets ratio. Again the authors underpinned the need for additional high quality research in the field. In a prospective cohort of 55 patients, Chidester et al. found that coagulopathy was a predictor of initiation of the protocol and fewer thromboembolic complications were observed in the patients who received massive transfusion.

### Permissive resuscitation

Permissive or low volume resuscitation is a concept that has evolved over the last years in the management of adult trauma patient and has gained considerable attention both in clinical setting and the related basic research [16–19]. This practice has not gained wide acceptance in the management of paediatric patients and some authors advocate extreme vigilance, questioning the non-validated theoretical benefits of such an approach in children [20].

### Coagulopathy and tranexamic acid

Trauma-induced coagulopathy is one of the most commonly encountered complications of trauma during the resuscitation process [21]. The main causative factor behind the development of coagulopathy is the local activation of the coagulation system after trauma and the subsequent acidosis, hypothermia, hemodilution and coagulation factor consumption. Current thinking in the field suggests that the basic mechanisms of this phenomenon differ between the adults and children. The two main potential mechanisms in the trauma-induced coagulopathy in paediatric population include the so-called acute traumatic coagulopathy and the iatrogenic coagulopathy. Without the undisputable evidence available, it is thought that in children the former pathway is mediated by activation of protein C, glycocalyx shedding and degradation of Weibel–Palade body. Nevertheless, the classic factors (hypothermia, volume depletion, hemodilution and coagulation factor depletion) are considered as the main mechanisms of coagulopathy [22].

Coagulopathy in paediatric trauma is of paramount importance and there are recent reports in the literature suggesting that it is associated with mortality. Hendrickson et al. [23], in a study evaluating the prevalence of coagulopathy of 102 traumatised children presented to the emergency department of a level-2 trauma centre, concluded that almost half of the studied patients were coagulopathic during the first 24 h and that was related to increased mortality and morbidity (longer intensive care stay and number of days requiring ventilation). The authors stressed the fact that more research is needed to clarify the role of coagulation factor replacement and massive transfusion practice. Moreover, Whittaker et al. [24] retrospectively reviewed 803 patients with severe trauma admitted in Level-1 civilian trauma centre. Early coagulopathy was correlated with significant increase in mortality when associated with traumatic brain injury. The authors suggested that early correction of coagulopathy could lead to substantial decrease in mortality.

The safety and efficiency of tranexamic acid in adult trauma has been well documented [25, 26] and its use as a first line medication is established and considered a “standard practice” [27]. The use of tranexamic acid in perioperative setting in children has been studied in cardiac [28, 29], spinal [30, 31] and craniofacial [32, 33] surgery. Beno et al. [34] in a narrative review of the contemporary literature concluded that there is lack of strong evidence to support its use in adolescent trauma resuscitation. On the other hand, the authors suggested that its use is likely to be beneficial in paediatric trauma, given the robust clinical evidence of its use in adult resuscitation, its proven safety and efficacy in other fields of paediatric surgery and its documented cost-effectiveness. Nevertheless, the authors recognized that further research is necessary in the filed to substantiate their suggestions. Additionally in a retrospective review of 766 paediatric patients presented in a four year period (2008–2012) to the North Atlantic Treaty Organization Role 3 hospital, Camp Bastion, Afghanistan, Eckert et al. [35] reported on the factors related to tranexamic acid use and mortality. Tranexamic acid was used in 10 % of the cases and especially in cases with severe abdominal and extremity trauma. The use of tranexamic acid was independently associated with increased mortality and no adverse safety or medication-related complications (i.e. cardiovascular or other thromboembolic phenomena) were observed.

## Education

Simulation is an emerging filed of training [36–39] and it has been used in neonatal resuscitation, paediatric advanced life support, procedural skills training and crisis resource management training [40]. In a study evaluating the use of simulation and script debriefing, Cheng et al. concluded that learning and team leader behavioral performance during simulated cardiopulmonary arrests is improved with the use of standardized script by novice instructors who facilitate team debriefing. Cole at al [41] performed a randomized controlled trial comparing two provider-endorsed manual paediatric fluid resuscitation techniques in a simulated setting. The authors tested the rate of fluid administration using a model simulating a 5 kg child in decompensated shock. Based on the results of their study the authors suggested that the connect–reconnect technique might be more efficient in situations necessitating rapid resuscitation thus demonstrating how the results of comparing two basic techniques in a simulated model could be extrapolated in clinical scenarios. Notwithstanding the latest advances, simulation resuscitation training is still in its infancy and future research is required to determine its effectiveness and clarify its role in training [36]. Along the same line e-learning might be a useful adjunct in the resuscitation related training process [42].

## Summary

Substantial advances have been made over the last five years in most of the fields of paediatric trauma resuscitation. Evidence suggests that too vigorous fluid might be harmful for children. Permissive resuscitation is not well accepted in the paediatric population. Massive transfusion practice is gaining popularity in cases of severe hemorrhagic shock. Trauma-related coagulopathy in children constitutes a major factor contributing to mortality and should be taken into consideration in paediatric physiology. Up-to-date there is no robust evidence for the use of tranexamic acid in severe paediatric trauma but current expert opinion suggests it use. The role of simulation in resuscitation training has been emphasized and promoted by many authors. Future directions in research should incorporate the above to support meaningful progress in the field.
